# Rheological and biochemical comparison of cord and adult blood red cells for transfusion applications

**DOI:** 10.1038/s41598-026-42457-4

**Published:** 2026-03-13

**Authors:** Larysa Mykhailova, Cristina Vercellati, Tiziana Montemurro, Anna Zaninoni, Anna Marcello, Elisa Fermo, Alessandro Cherubini, Silvia Cimoni, Paola Bianchi, Daniele Prati, Stefania Villa

**Affiliations:** 1 Department of Transfusion Medicine and Hematology, Fondazione IRCCS Ca’ Granda Ospedale Maggiore Policlinico, Milan, Italy; 2 Hematology Unit, Pathophysiology of Anemias Unit, Fondazione IRCCS Ca’ Granda Ospedale Maggiore Policlinico, Milan, Italy; 3 Precision Medicine-Biological Resource Center and Department of Transfusion Medicine, Fondazione IRCCS Ca’ Granda Ospedale Maggiore Policlinico, Milan, Italy; 4 Milano Cord Blood Bank and Department of Transfusion Medicine, Fondazione IRCCS Ca’ Granda Ospedale Maggiore Policlinico, Milan, Italy

**Keywords:** Cord blood, Red blood cells, Deformability, Rheological properties, Preterm transfusions, Ektacytometer, Biochemistry, Cell biology, Medical research, Physiology

## Abstract

Cord blood (CB) is a promising alternative source of red blood cells (CB-RBC) for neonatal transfusion due to their high fetal hemoglobin content and potential physiological benefits in preterm infants, but their metabolic and rheological stability during storage and irradiation is not fully defined. This study examined whole cord blood (WCB) and processed CB-RBC stored up to 10 days (D10), with or without irradiation, and compared them with adult RBC (A-RBC) following neonatal transfusion guidelines. Ektacytometry showed significantly lower EImax in CB-RBC at day 0 (D0) and D10 (*p* < 0.05), while Omin and Ohyper remained stable. CB-RBC had lower ATP than A-RBC at baseline (2.8 ± 1.0 µmol/g Hb vs. 4.0 ± 0.3 µmol/g Hb) and after 10 days (2.0 ± 1.1 µmol/g Hb vs. 3.0 ± 1.9 µmol/g Hb). Hemolysis in CB-RBC was minimal (median 0.05, range 0.04–1.17% at D0 vs. median 0.19, range 0.16–1.23% at D10), and residual WBC content met limits (< 1 × 10⁶). Storage induced expected biochemical changes in both CB-RBC and A-RBC, including increased potassium, pO2 and lactate, decreased sodium and glucose, and pH decline, though D10 pH and potassium evaluation in CB-RBC was partly affected by hemolysis. Irradiation produced minimal effects on ATP and rheology. Overall, CB-RBC preserved acceptable metabolic and rheological properties during short-term storage and irradiation, supporting further exploration of their suitability for neonatal transfusion.

**Supplementary Information:**

The online version contains supplementary material available at 10.1038/s41598-026-42457-4.

## Introduction

Adult blood red cells (A-RBC) have been extensively characterized in terms of origin, structure, and function^1^. They derive from hematopoietic stem cells (HSCs) in the bone marrow, where erythropoietin regulates enucleation and cytoplasmic remodeling to form mature, biconcave cells^2^. Their deformability, crucial for oxygen transport through narrow capillaries, depends on the dynamic interactions between the plasma membrane and the spectrin-actin cytoskeleton^3^.

Hemoglobin composition distinguishes A-RBC from cord blood (CB)-RBC: adult hemoglobin (HbA, α_2_β_2_) replaces fetal hemoglobin (HbF, α_2_γ_2_) after birth^4^. At delivery, HbF accounts for about 80% of total hemoglobin but progressively decreases as HbA synthesis increases during infancy^5–7^. HbF exhibits higher oxygen affinity than HbA due to weaker binding to 2,3-diphosphoglycerate (2,3-DPG), optimizing placental oxygen transfer^8^.

These differences have major implications in neonatal transfusion medicine, particularly for extremely low gestational age neonates (ELGANs), whose immature antioxidant and homeostatic systems increase susceptibility to oxidative stress and related morbidities such as retinopathy of prematurity (ROP), bronchopulmonary dysplasia (BPD), and necrotizing enterocolitis (NEC)^9–12^. The early HbF-to-HbA transition, combined with the high transfusion burden and small circulating blood volume, further heightens transfusion-related risks in this fragile population^10,13^.

CB has long been valued for its rich HSC content and distinctive RBC properties. Since the first report by Broxmeyer et al. (1989) demonstrating its potential for hematopoietic reconstitution^14^, over 800,000 CB units have been banked and more than 55,000 transplants performed worldwide^15^. Despite the increasing use of haploidentical transplantation, CB remains a critical alternative for patients lacking matched donors, particularly in pediatric or high-risk cases^16,17^. Yet, fewer than 10% of collected CB units meet transplantation criteria, leaving many clinically safe but underutilized^18^.

Recent studies have suggested that CB-RBC may offer transfusion advantages over A-RBC, owing to their high HbF content, distinct membrane structure, and metabolic profile, which confer enhanced oxygen affinity and potential immunomodulatory effects^19–22^. These features support the hypothesis that CB-RBC could serve as a safer and more physiologically compatible transfusion source for neonates, especially ELGANs.

However, stored RBC— both CB-RBC or A-RBC —undergo progressive biochemical and morphological deterioration known as “storage lesions,” including oxidative damage, loss of deformability, and metabolic depletion^23–25^. These changes, accentuated by reactive oxygen species accumulation, lipid peroxidation, and ATP depletion, reduce post-transfusion survival and functional efficacy^7,26^. This issue is particularly critical in neonates, who are more sensitive to oxidative imbalance and transfusion stress.

It’s well known that leukoreduction and irradiation mitigate some immune risks, on the contrary, their effect on CB-RBC quality during storage remains poorly defined. To date, the effects of storage and irradiation on the rheological, and biochemical integrity of CB-RBC have not been comprehensively investigated. To address this gap, the present study sought to characterize whole CB (WCB) and evaluate the stability of key functional properties in processed CB-RBC for up to 10 days following treatment and irradiation. A secondary aim was to compare these features with those of A-RBC to determine their suitability for neonatal—particularly preterm—transfusion, in alignment with current evidence-based guidelines^42^.

## Results

### Characterization of whole cord blood and comparison with whole adult blood

#### Physiological properties

**Fig. 1:**
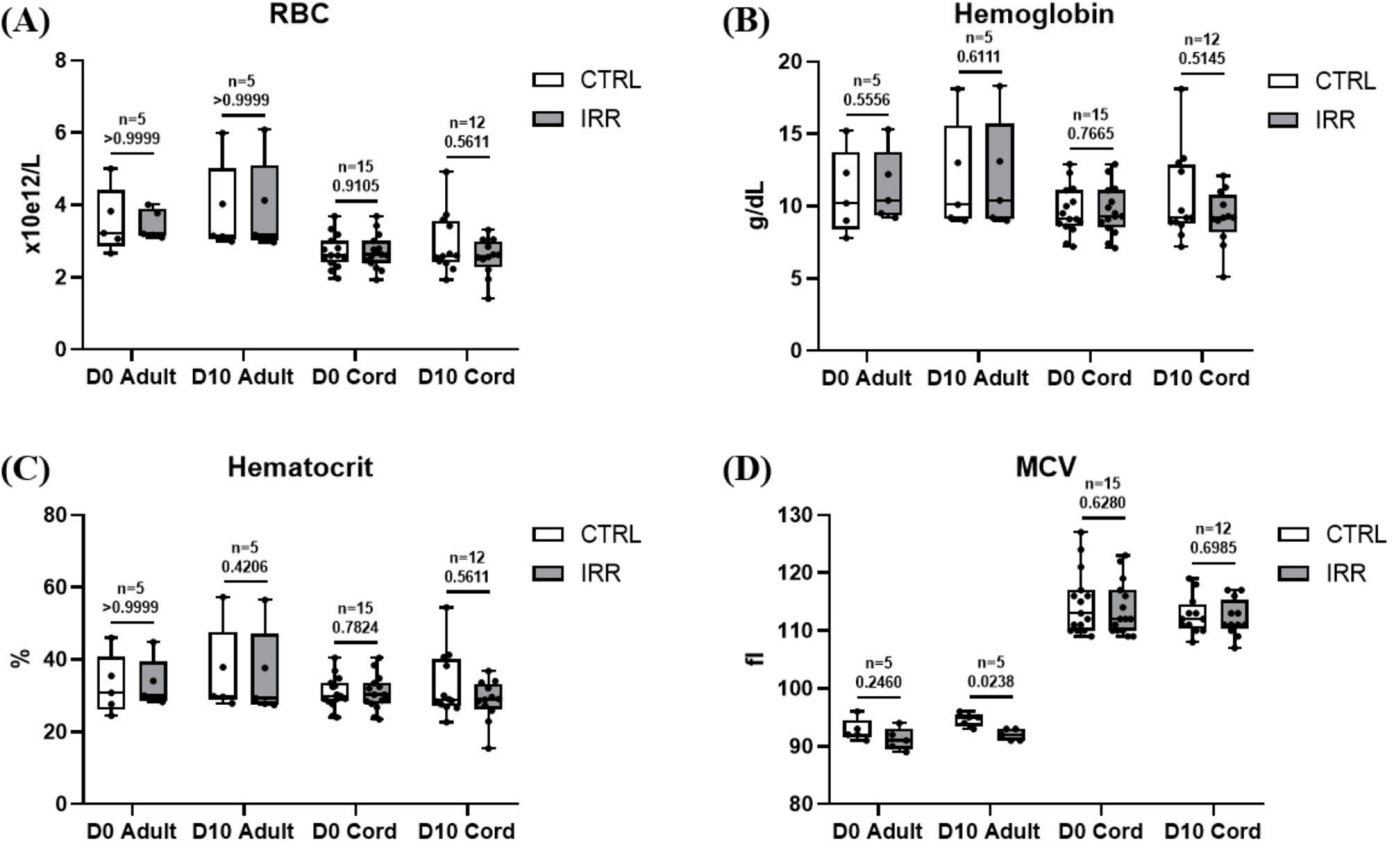
Hematological properties of whole adult blood and whole cord blood. All data were reported as box and whisker plots, where the line in the middle of the box represents the medians, tops and bottoms of the boxes the 25th and 75th quartiles, respectively, and the whiskers the minimum to maximum value. Nonparametric Mann-Whitney test was performed. RBC: Red Blood Cells; MCV: Mean Cellular Volume; CTRL: control sample; IRR: irradiated sample; D0: day 0 of storage; D10: day 10 of storage.

Physiological analyses revealed that no statistically significant difference was detected in RBC count between whole adult blood (WAB) and WCB units. Neither irradiation nor storage substantially affected total RBC numbers (Fig. 1A). Hemoglobin content remained unchanged after irradiation and during storage (Fig. 1B). Hematocrit levels were comparable between WAB and WCB units and were not influenced by irradiation or storage (Fig. 1C). In contrast, mean cell volume (MCV) differed significantly between WAB and WCB samples both at day zero of storage (D0 – median: 92.1, range 90.5–95.7 fL vs. 112.7, range 108.8-126.5 fL, *p* < 0.001) and after 10 days of storage (D10 – median: 94.6, range 93-95.7 fL vs. 112, range 106.2-119.3 fL) (Fig. 1D).

#### Rheological properties

**Fig. 2:**
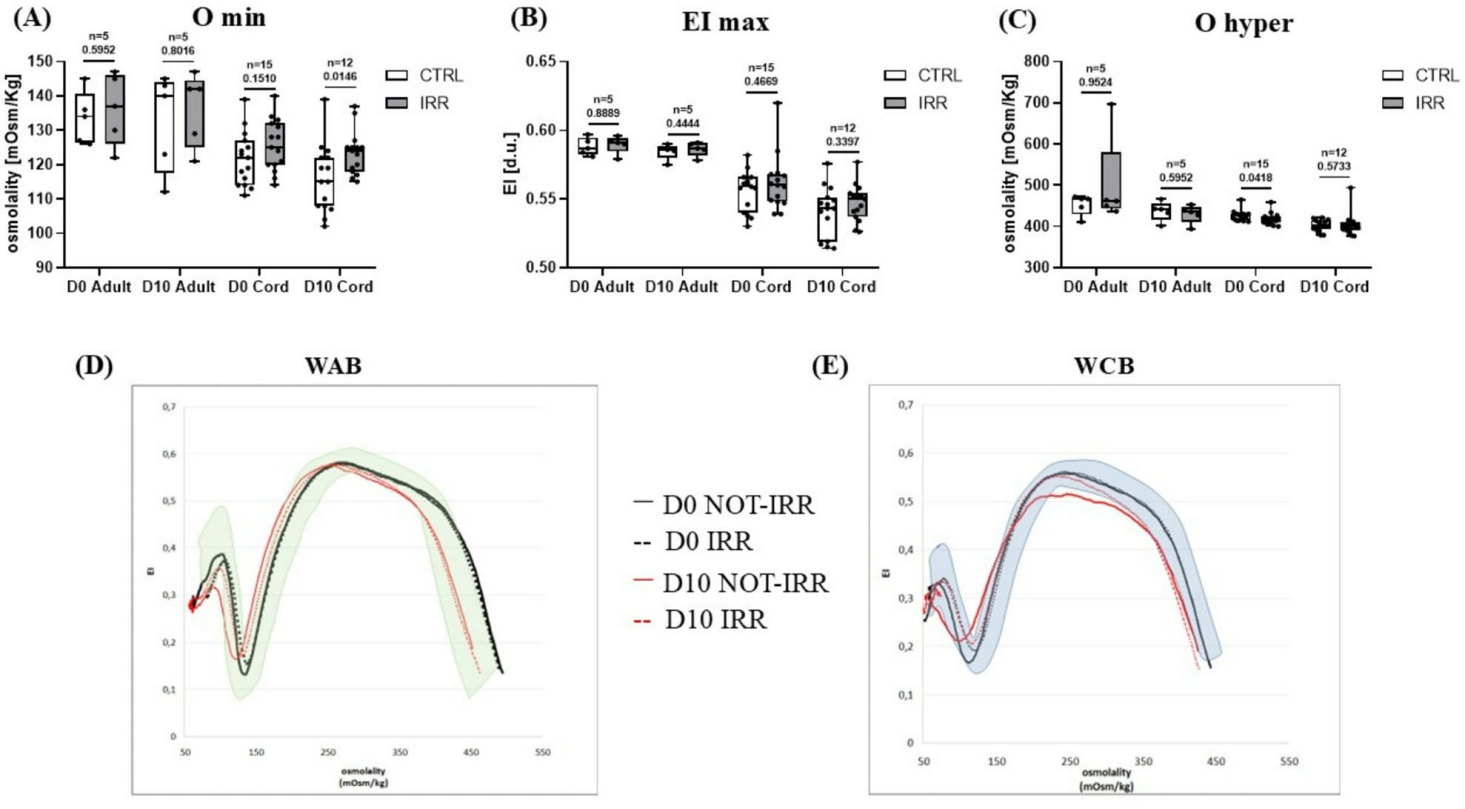
Rheological properties of whole adult blood (WAB) and whole cord blood (WCB) units and an example of ektacytometric curve. All data were reported as box and whisker plots, where the line in the middle of the box represents the medians, tops and bottoms of the boxes the 25th and 75th quartiles, respectively, and the whiskers the minimum to maximum value. Nonparametric Mann-Whitney test was performed. Omin: indicative of osmotic fragility; EI max: maximum deformability; Ohyper: indicative of cellular hydration; CTRL: control sample; IRR: irradiated sample; D0: day 0 of storage; D10: day 10 of storage. Shaded area corresponds to normal reference range, dotted line corresponds to irradiated cells (IRR); continuous line corresponds to not-irradiated cells (NOT-IRR), D0 = black line; D10 = red line.

Ektacytometric analysis showed that all the five WAB samples stored in bags exhibited reference curves comparable to those of peripheral blood used as normal control in each analysis. A reference range for CB was established from the ten samples analyzed at D0 (see Supplementary Materials, Figure S1). No significant differences in Omin (indicative of osmotic fragility) were detected between WAB and WCB units. Irradiation and storage did not produce significant changes in Omin value (Fig. 2A). The maximum elongation index (EImax) was significantly higher in WAB (median: 0.59, range 0.58–0.60 EI d.u for both irradiated and non-irradiated samples at D0; median: 0.59, range 0.58–0.59 EI d.u. for both irradiated and non-irradiated samples at D10) units compared to WCB samples (median: 0.56, range 0.56–0.58 EI d.u for non-irradiated samples and median: 0.56 range 0.56–0.68 EI d.u. for irradiated samples at D0; median: 0.54, range 0.54–0.58 EI d.u. for non-irradiated samples and median: 0.55, range 0.55–0.58 EI d.u. for irradiated samples at D10). The statistical EImax comparison between WAB and WCB yielded a p-value of < 0.003, indicating more evident deformability in WAB samples. Irradiation does not cause changes to the EImax value (Fig. 2B). Ohyper (indicative of cellular hydration) remained stable across all conditions, with no significant differences between WAB and WCB or in response to irradiation or storage (Fig. 2C). These findings were consistent with the curves obtained by ektacytometric analysis (Fig. 2D–E).

#### RBC morphology

**Fig. 3:**
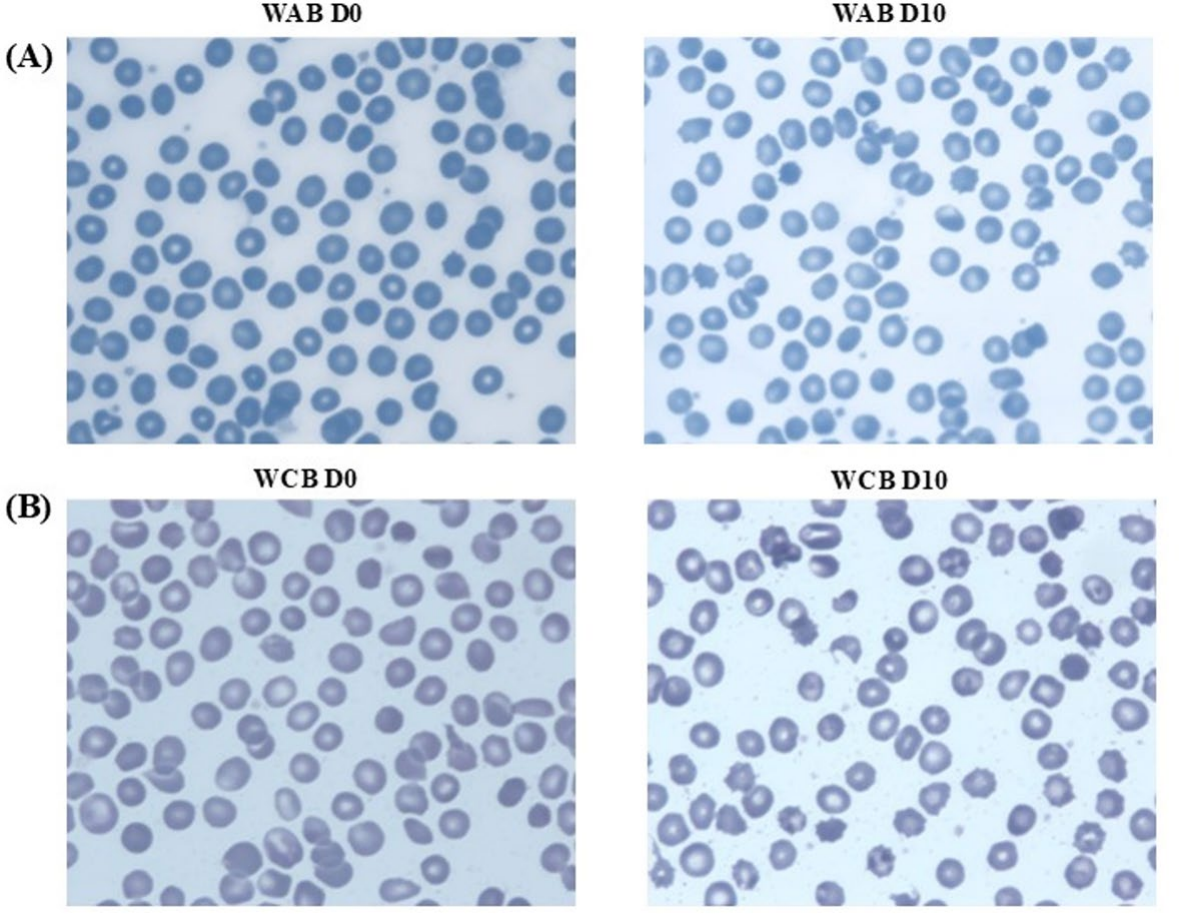
A representative micrograph of whole adult blood cells (WAB) and whole cord blood cells (WCB) morphology at day zero of storage (D0) and at day ten of storage (D10).

All analysed WAB and WCB samples showed moderate to marked anisopoikilocytosis both on D0 and D10 before and after irradiation (Fig. 3). The morphology on D0 and D10 of storage, showed a low percentage of stomatocytes, spherocytes, and schistocytes under all conditions. As expected, a marked increase in echinocytes was observed after ten days of storage (see Supplementary Materials, Table S1).

### Determination of ATP levels in whole cord blood

Fresh WCB samples exhibited ATP concentrations consistent with the established reference range for WAB (3.6–4.9 µmol/g Hb). During storage, in non-irradiated samples (*n* = 16), the mean ATP content decreased from 4.1 ± 0.9 µmol/g Hb at D0 to 2.9 ± 0.9 µmol/g Hb at D10, (paired t = 3.2, *p* = 0.005). In contrast, in irradiated samples ATP level decreased from 3.7 ± 1.1 µmol/g Hb at D0 to 3.1 ± 1.2 µmol/g Hb at D10 (paired t = 1.4, *p* = 0.19). Overall, these findings indicate that storage induces a more pronounced decline in ATP content in non-irradiated samples, while irradiation appears to partially mitigate ATP loss over time.

### Characterization of cord bood red cells and comparison with adult red blood cells

#### Physiological properties

**Fig. 4:**
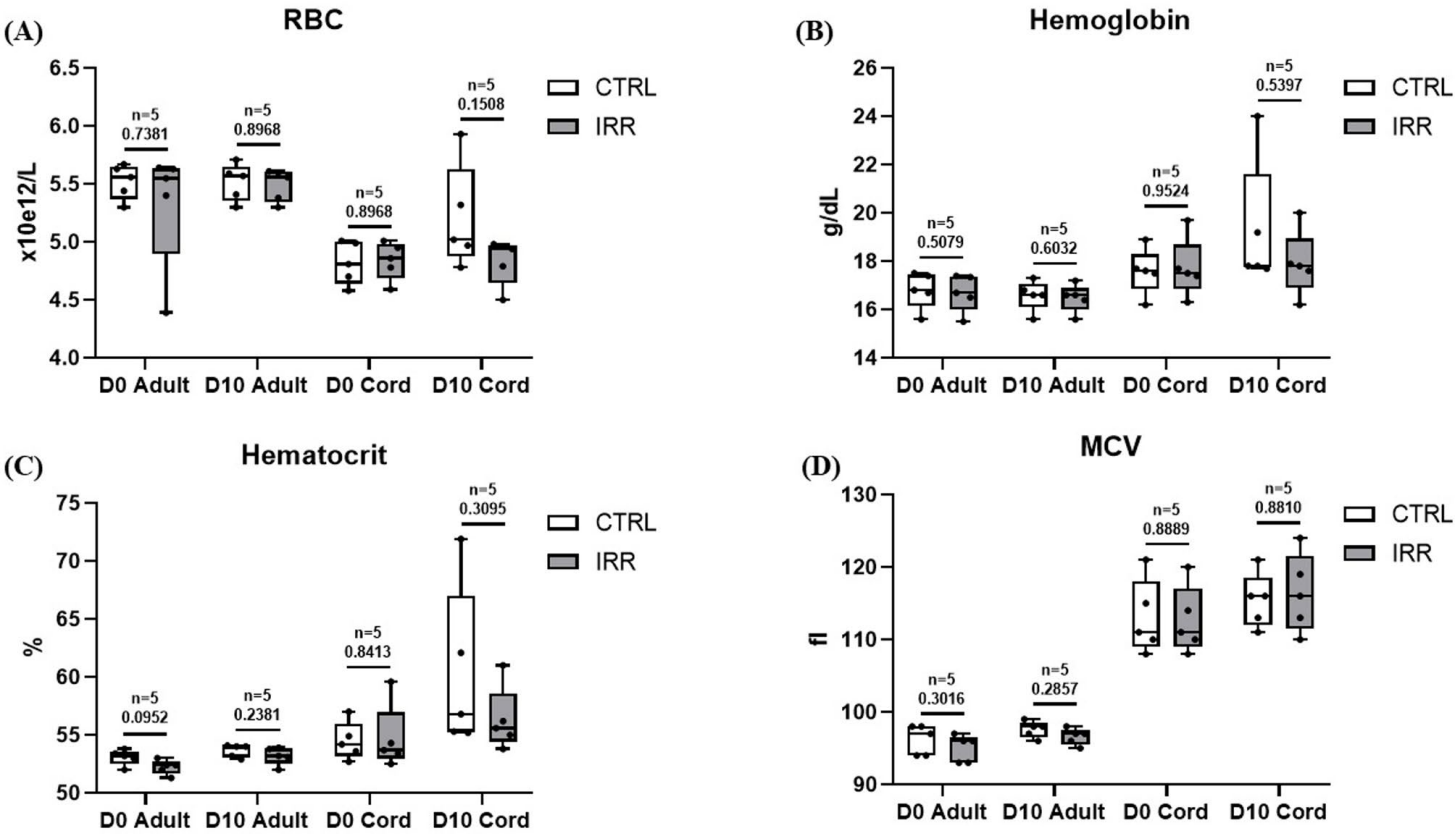
Hematological properties of adult red blood cells (A-RBC) and cord blood red cells (CB-RBC). All data were reported as box and whisker plots, where the line in the middle of the box represents the medians, tops and bottoms of the boxes the 25th and 75th quartiles, respectively, and the whiskers the minimum to maximum value. Nonparametric Mann-Whitney test was performed. RBC: Red Blood Cells; MCV: Mean Cellular Volume; CTRL: control sample; IRR: irradiated sample; D0: day 0 of storage; D10: day 10 of storage.

To evaluate the transfusion potential of WCB, we compared CB-RBC and A-RBC units prepared under the same conditions used in clinical practice. Physiological analyses revealed no significant effects either at the time of irradiation or during subsequent storage of RBC at D0 and D10 (Fig. 4A). Hemoglobin content and hematocrit levels remained comparable between groups and were unaffected by storage or irradiation (Fig. 4B, C). In contrast, MCV differed significantly between A-RBC (median: 96.8, range 94.1–98.1 fL for non-irradiated samples and median: 96, range 92.9–97 fL for irradiated samples at D0; median: 98, range 95.5–99 fL for non-irradiated samples and median: 97, range 94.7–98 fL for irradiated samples at D10) and CB-RBC units (median: 111.4, range 108–121 fL for non-irradiated samples and median: 111, range 108–120 fL for irradiated samples at D0; median: 115.7, range 111–121 fL for non-irradiated samples and median: 116.1, range 110–124 fL for irradiated samples at D10) at both D0 and D10, in control and irradiated samples. The statistical MCV comparison between A-RBC and CB-RBC yielded a p-value of = 0.0079 (Fig. 4D).

#### Rheological properties

**Fig. 5:**
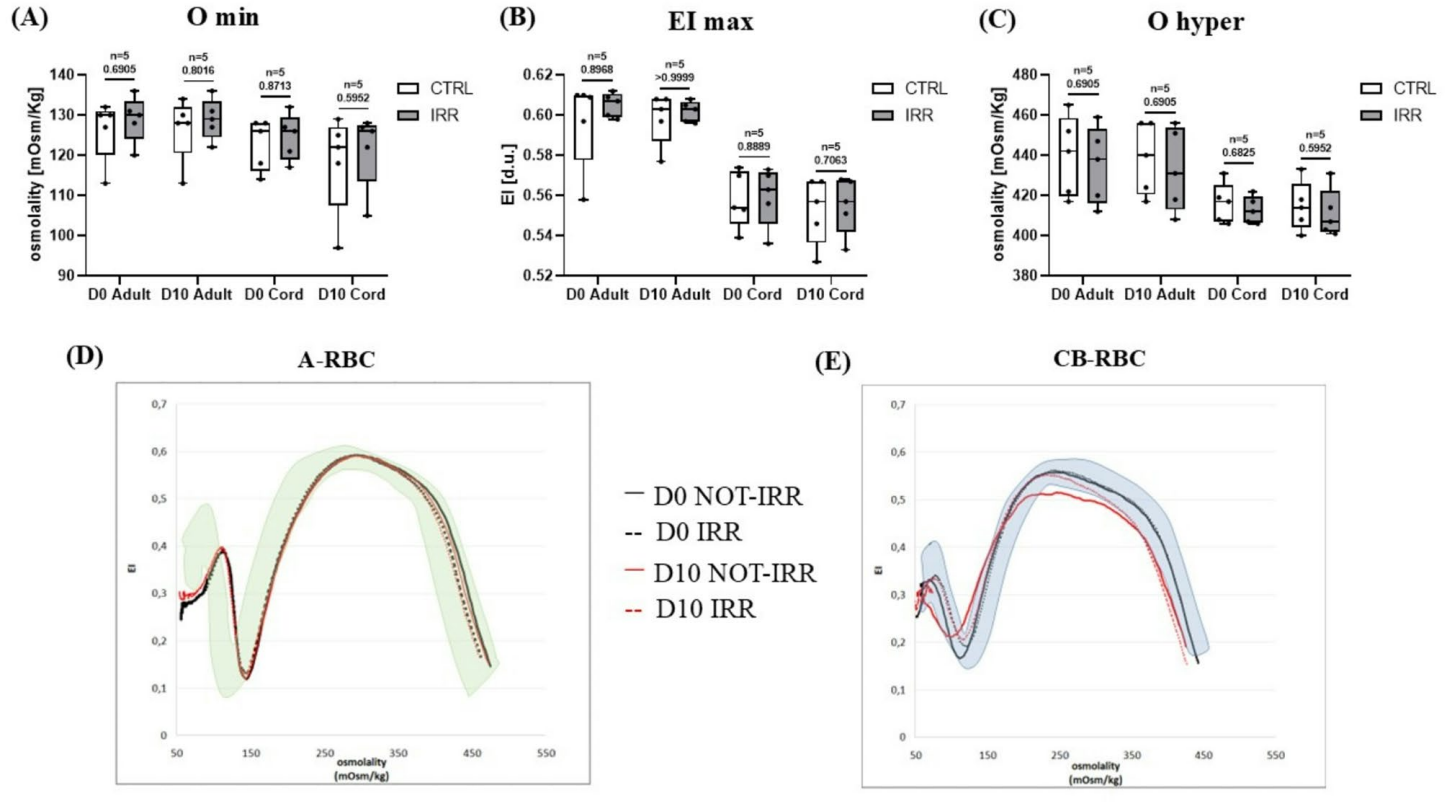
Rheological properties of adult red blood cells (A-RBC) and cord blood red cells (CB-RBC) units and an example of ektacytometric curve. All data were reported as box and whisker plots, where the line in the middle of the box represents the medians, tops and bottoms of the boxes the 25th and 75th quartiles, respectively, and the whiskers the minimum to maximum value. Nonparametric Mann-Whitney test was performed. Omin: indicative of osmotic fragility; EI max: maximum deformability; Ohyper: indicative of cellular hydration; CTRL: control sample; IRR: irradiated sample; D0: day 0 of storage; D10: day 10 of storage. Shaded area corresponds to normal reference range, dotted line corresponds to irradiates cells (IRR); continuous line corresponds to not-irradiated cells (NOT-IRR), D0 = black line; D10 = red line.

No significant difference in Omin was observed between A-RBC and CB-RBC. Irradiation increased Omin in both groups, while no statistically significant difference detected after storage (Fig. 5A). EImax was significantly higher in A-RBC (median: 0.61, range 0.56–0.61 EI d.u. for non-irradiated samples and median: 0.61, range 0.60–0.61 EI d.u. for irradiated samples at D0; median: 0.60, range 0.58–0.60 EI d.u. for non-irradiated samples and median: 0.60, range 0.60–0.61 EI d.u. for irradiated samples at D10) than in CB-RBC (median: 0.53, range 0.56–0.57 EI d.u. for both irradiated and non-irradiated samples at D0; median: 0.55, range 0.54–0.57 EI d.u. for non-irradiated samples and median: 0.56, range 0.54–0.57 fL for irradiated samples at D10) at both D0 and D10 with no changes following irradiation or storage. The statistical EImax comparison between A-RBC and CB-RBC yielded a p-value of < 0.05 (Fig. 5B). Ohyper remained stable under all conditions (Fig. 5C). These results were consistent with the ektacytometric profiles shown in Fig. 5D–E.

### Determination of ATP levels in cord and adult red blood cells

At D0, CB-RBC exhibited lower ATP levels than A-RBC, and irradiation did not significantly affect ATP content in either group. During storage, in non-irradiated CB-RBC (*n* = 5) the mean ATP concentration decreased from 2.8 ± 1.0 µmol/g Hb at D0 to 2.0 ± 1.1 µmol/g Hb at D10 (paired t = 2.9, *p* = 0.045). In irradiated CB-RBC, ATP levels were 2.5 ± 1.0 µmol/g Hb at D0 and 2.2 ± 1.1 µmol/g Hb at D10, showing no significant change (paired t = 0.8, *p* = 0.46). In non-irradiated A-RBC (*n* = 5), ATP concentrations decreased from 4.0 ± 0.3 µmol/g Hb at D0 to 3.0 ± 1.9 µmol/g Hb at D10 (paired t = 1.3, *p* = 0.27). Similarly, in irradiated A-RBC, values changed from 4.1 ± 0.5 µmol/g Hb at D0 to 2.9 ± 1.8 µmol/g Hb at D10 (paired t = 1.2, *p* = 0.30). Overall, these data indicate that ATP content decreases during storage in both CB-RBC and A-RBC, but the decline reaches statistical significance only in non-irradiated CB-RBC. Irradiation under the tested conditions did not significantly affect ATP preservation in either group.

### Evaluation of red blood cells storage-related biochemical changes

To evaluate storage-related changes, additional biochemical parameters were measured in A-RBC and CB-RBC units at D0 and D10 (Table 1, all parameters expressed as median and range). Residual WBC (white blood cells) counts remained within regulatory limits (< 1 × 10^6^) for both types. CB-RBC showed an increase in hemolysis during storage, however remaining within legal limits (< 0.8%). Potassium levels rose in both components; however, accurate measurement in CB-RBC at D10 was limited by hemolysis interference (the only value obtained after measurement was > 15, a precise quantitative determination could not be achieved). Sodium and glucose levels decreased over 10 days of storage, accompanied by increased lactate. pH declined during storage, though precise values for CB-RBC at day 10 could not be determined due to hemolysis (the only value obtained after measurement was < 6.5). Finally, pO_2_ values increased over the 10-day storage period in both CB-RBC (*p* = 0.008, medians and ranges are shown in Table 1) and A-RBC (*p* = 0.008, medians and ranges are shown in Table 1) samples. Moreover, a clear difference between the two blood sources was observed: CB-RBC exhibited significantly higher pO_2_ levels than A-RBC at both D0 (*p* = 0.008) and D10 (*p* = 0.008) medians and ranges are shown in Table 1.

**Table 1:** Biochemical analysis of stored CB-RBC (cord blood red cells) and A-RBC (adult blood red cells).

	*N*.	CB-RBC			A-RBC		
		D0	D10	*p*-value	D0	D10	*p*-value
Hemolysis (%)	5	0.05 (0.04–1.17)	0.19 (0.16–1.23)	0.0356	0.12 (0.08–0.23)	0.23 (0.03–0.55)	0.249
Rest WBC (X10^6^)	5	0.05 (0.01–0.08)	N.D.	-	0.05 (0–0.09)	N.D.	-
Potassium (mmol/L)	5	2.2 (1.9–3.1)	> 15	-	3.35 (3–3.5)	21.6 (20.6–25)	0.01219
Sodium (mmol/L)	5	146.4 (143.4–148.5)	115.6 (110.7–118.4)	0.01219	140.3 (139.5–141.8)	134 (132–137)	0.01193
Glucose (mmol/L)	5	38.9 (37.3–40.7)	36.4 (32–38.2)	0.0601	30.5 (29.6–32)	26.2 (24.9–27.4)	0.01219
Lactate (mmol/L)	5	4.6 (3.5–6.2)	10.9 (10.9–11.3)	0.01219	2.7 (2.6–2.8)	7.3 (6.6–9.3)	0.01219
pH	5	6.6 (6.6–6.7)	< 6.5	-	6.9 (6.8–6.9)	6.6 (6.5–6.7)	0.01141
pO_2_	5	122.6 (137.4–208.2)	255.9 (249–266.6)	0.008	44.5 (43.4–48.1)	60.7 (59.6–73.9)	0.008

D0: day 0 of storage; D10: day 10 of storage; Rest: residual; N.D.: not done. Values are expressed as median (range). Nonparametric Mann-Whitney test was performed.

## Discussion

Neonatal guidelines generally prioritize irradiated A-RBC, although CB-RBC are increasingly viewed as a promising alternative for preterm infants, with potentially lower risks of complications and alloimmunization^30–32^. A central challenge remains the metabolic deterioration that accompanies RBC storage, despite ongoing improvements in preservation strategies^25,33^.

This study (March 2022–December 2024) aimed to characterize WCB in comparison with WAB and to assess whether CB-RBC display physiological and qualitative features comparable to A-RBC for neonatal transfusion, with particular relevance for ELGANs, who maintain HbF production and are highly susceptible to transfusion-related morbidities such as ROP and bronchopulmonary dysplasia when exposed to adult RBC^34^.

WCB exhibited storage and post-irradiation profiles largely consistent with WAB, with differences in deformability and elasticity likely associated with a higher proportion of echinocytes and erythroblasts, typical indicators of storage-related osmotic or oxidative stress^25,27^. CB-RBC showed physiological parameters, residual WBC counts, and rheological behaviour comparable to A-RBC, with slightly lower but acceptable ATP values, consistent with Yoshida et al.^22^. Both RBC types demonstrated the expected decline in pH during storage; however, pH values below 6.5 could not be measured in some CB-RBC units, this limitation could be attributable to the specific characteristics of CB-RBC. These observations further emphasize the need to transfuse CB-RBC units within a significantly shorter time frame than A-RBC units.

Metabolic changes—rising lactate, decreasing glucose—reflected anaerobic glycolysis during storage^12,23,33^. Across the 10 days of storage, including post-irradiation analyses, both products developed classical storage lesions (elevated potassium, hemolysis, lactate), known contributors to inflammation and organ dysfunction in preterm infants^35,36^. Despite progressive increases, extracellular potassium and hemolysis in CB-RBC remained within acceptable limits^28^. Current recommendations—transfusion rates of 0.5 mL/kg/min^37,38^, use of units stored < 7 days for high-volume transfusions^41^, and irradiated units within 14 days^28,39,43,44^—remain appropriate for both products. Current recommendations—transfusion rates of 0.5 mL/kg/min^37,38^, use of units stored < 7 days for high-volume transfusions^41^, and irradiated units within 14 days^28,39,43,44^—remain appropriate for both adult and cord CB–RBC units. Potassium levels represent an important consideration in neonatal transfusions, given the high susceptibility of neonates to hyperkalemia due to their low total blood volume and immature renal function. In our study, potassium concentrations in A-RBC units increased from 3.35 to 21.6 mM over the storage period, whereas in CB-RBC potassium rose from 2.2 mM to values beyond the measurable range of the analyzer (only detected value > 15mM). This difference and the impossibility of obtaining accurate measurements after storage reflects the intrinsic properties of CB-RBC, including altered membrane stability, higher membrane rupture rates, and modified cellular morphology, which collectively influence ion leakage during storage^45^. These observations underscore the importance of closely monitoring potassium load and adhering to recommended storage durations and transfusion rates to minimize the risk of hyperkalemia while ensuring effective oxygen delivery and compatibility with the neonatal microcirculation.

In relation to the higher pO_2_ values observed in CB-RBC units compared with A-RBC units may be explained by intrinsic physiological differences between fetal and adult erythrocytes. CB contains a high proportion of HbF, which is characterized by a higher affinity for oxygen and a left-shifted oxygen dissociation curve due to its reduced interaction with 2,3-bisphosphoglycerate^46^. As a consequence, HbF achieves higher oxygen saturation at lower pO_2_ levels compared with adult hemoglobin, which may contribute to higher measured pO_2_ values in CB units following collection and processing.

Rheologically, CB-RBC exhibited lower deformability and higher MCV values than A-RBC at baseline; following storage or irradiation, these differences remained largely comparable to baseline. The increased MCV of CB-RBC reflects intrinsic cellular size differences that contribute to their biomechanical behavior and are associated with a higher resistance to deformation rather than enhanced deformability. These findings are in line with previous reports describing subtle but consistent mechanical differences between cord and A-RBC, which may have functional implications in the neonatal setting^26^. Moreover, despite these rheological differences, recent clinical evidence indicates that CB-RBC transfusions may be associated with favorable cerebral tissue oxygenation in preterm neonates compared with A-RBC, supporting their physiological compatibility in neonatal transfusion practice^40^. Notably, CB-RBC exhibited lower hemolysis throughout storage, potentially limiting free hemoglobin and microvesicle release and offering a mechanistic rationale for improved neonatal outcomes. The metabolic and ionic shifts observed here may also underlie the physiological differences reported by Pellegrino et al. (2023)^40^, such as altered cerebral oxygen saturation and extraction, consistent with known effects of pH, pO_2_ and lactate on hemoglobin–oxygen affinity.

Studies comparing CB-RBC and A-RBC consistently report rheological differences that reflect physiological adaptation to the neonatal environment rather than enhanced deformability. In this context, Arbell et al. showed that CB-RBC exhibit rheological properties comparable to neonatal RBC, supporting their compatibility with neonatal microcirculation^26^. Similarly, the in vitro study by Dinara et al.^34^ demonstrated that CB-RBC preserve favorable rheological and metabolic characteristics during storage compared with A-RBC, indicating differences in cellular behavior rather than an absolute increase in deformability. These findings align with accumulating evidence that CB-RBC display distinct rheological profiles, primarily driven by intrinsic cellular features such as larger cell size (higher MCV) and specific membrane mechanics. As comprehensively reviewed by Pellegrino et al.^45^, neonatal and fetal RBC tend to exhibit greater resistance to deformation due to their increased volume, while membrane surface viscosity and hemoglobin viscosity remain largely comparable to those of A-RBC. Importantly, despite their larger size and lower deformability, effective microcirculatory flow in neonates is facilitated in vivo by lower plasma viscosity and the unique hemodynamic conditions of the neonatal circulation. In this context, the clinical observations reported by Teofili (2025)^41^, although not directly addressing storage-related biochemical parameters, are consistent with a preserved metabolic and structural profile of CB-RBC and support their suitability for neonatal transfusion. Taken together, these studies, in conjunction with our findings of stable rheological differences between CB-RBC and A-RBC during storage and irradiation, indicate that the potential advantage of CB-RBC does not rely on enhanced deformability, but rather on a distinct rheological and physiological profile - characterized by larger cell size, higher resistance to deformation, and storage stability - that is compatible with the low plasma viscosity and specific hemodynamic conditions of the neonatal circulatory system. This study has limitations, including a small and unbalanced sample size due to the difficulty of obtaining specific units, and missing pH and potassium measurements at D10 in some CB-RBC because of analytical interference. Finally, as an in vitro study, the clinical implications of these findings require confirmation through in vivo investigations.

## Conclusions

This study provides a detailed characterization of WCB and CB-RBC units, showing that they largely retain biochemical, morphological, and rheological properties comparable to WAB and A-RBC after collection, processing, and storage. Minor differences, such as slightly reduced deformability and higher MCV in CB-RBC, were observed but remained within physiological ranges, including after irradiation.

These findings indicate that CB-RBC could represent a physiologically suitable and qualitatively acceptable option for neonatal transfusion, particularly for preterm infants, including ELGANs. The limited accumulation of storage-related alterations over the 10-day period suggests that these units may be used safely within this timeframe. However, in vivo studies and clinical trials are required to confirm their safety, efficacy, and to better define optimal storage conditions and transfusion strategies.

## Materials and methods

### Blood units collection, selection and fractionation criteria

Fresh CB units were collected with maternal written consent and processed following Milan Cord Blood Bank (MICB) protocols. Units were stored and transported at 4°C and inspected upon arrival at MICB. Those deemed unsuitable for banking—based on low nucleated cell count (< 1.0 × 10⁹ cells/unit) and low volume (< 50 mL)—were directed for processing at the Transfusion Centre of Fondazione IRCCS Ca’ Granda Ospedale Maggiore Policlinico, Milan. Units selected for this study met specific inclusion criteria, including collection within 48 h of birth, absence of clots, net volume ≥ 50 mL, and nucleated cell counts above the threshold. All units underwent standard screening for infectious diseases.

Fractionation of WCB units was performed using the standardized protocol described by Samarkanova et al., 2023^25^, employing the CE-marked BioNest ABC fractionation kit (WhiteNestPharma S.r.l. Milan, Italy) for component separation. Following fractionation, the resulting CB-RBC units were leukoreduced using the BioNest EF (erythrocyte filtration) medical devices developed by WhiteNestPharma S.r.l. (Milan, Italy) and stored at 4 °C throughout the analysis period.

WAB units were obtained from healthy donors using donations that were ineligible for transfusion solely due to insufficient volume. These WAB units were processed according to the standard protocol of the Transfusion Centre, including fractionation and leukoreduction steps routinely used for adult blood components, and stored at 4 °C until analysis. All human samples were collected with written informed consent for research purposes and all experimental procedures were conducted according to the Italian law about the Regulation (EU) 2016/679 (General Data Protection Regulation) articles 5 and 11. All data were anonymous and for these reasons it was not necessary to receive the opinion of the Ethics Committee.

### Sample processing and analyses

Sixteen WCB were collected in Citrate Phosphate Dextrose Adenine (CPDA-1; JMS Singapore PTD, LTD, Republic of Singapore) and processed to assess RBC quality and functionality. Following fractionation, a 10 mL aliquot was drawn from each unit and divided into two portions: one subjected to gamma irradiation and the other analyzed fresh to evaluate freshly collected RBC. The remaining fraction of each unit was stored in the final collection bag under standard conditions at 4 °C. On day D10, a second aliquot (10 mL) was drawn from initial unit and similarly divided into irradiated and non-irradiated samples for subsequent analyses. All tests were performed immediately after irradiation process both at D0 and D10. Biochemical activity was assessed through ATP quantification, while rheological properties were determined using an ektacytometer (laser-assisted optical rotational cell analyzer) to measure RBC deformability. Morphological features were examined by light microscopy, and hematological parameters were characterized through complete blood counts. For comparative purposes, five WAB obtained from healthy adult donors in Citrate Phosphate Dextrose (CPD) were analyzed in parallel using the same assays, allowing a direct comparison of RBC quality and functionality between cord and adult whole blood.

In a subsequent phase, RBC concentrates were obtained from leukoreduction and fractionation independently from five CB-RBC units and five A-RBC collected from healthy adult donors; these concentrates were not derived from the previously analyzed WCB or WAB units. The same set of analyses applied to WCB and WAB was performed on these RBC concentrates, including assessment of ATP content, RBC deformability, and morphology. Additional evaluations in the RBC concentrates included residual white blood cell counts, hemolysis, and biochemical measurements of potassium, sodium, glucose, lactate, pO_2_ and pH.

### Quality test evaluation

Hematological analysis was conducted using the Sysmex XN-1000 system, which measures erythrocytes, leukocytes, Hb, platelet count, hematocrit, and leukocyte formula. Flow cytometry analysis was conducted at the Interdepartmental Centre for Flow Cytome-try and Experimental Hepatology at Fondazione IRCCS Ca’ Granda Policlinic of Milan, focused on counting residual WBCs after CB-RBC filtration. Results were expressed as the absolute number of leukocytes per µL of sample. Potassium, sodium, glucose, lactate, pO_2_ and pH values were determined using the automated analyser RAPIDPoint^®^ 500e Blood Gas System (Siemens Healthineers, Erlangen, Germany). The determination was performed on a 3 mL sample of AB/CB-RBC collected in a heparinized syringe (Smiths Medical ASD, Inc., Minneapolis, USA). To assess hemolysis in post-fractionated CB-RBC, a 1 mL sample was first analysed with a complete blood count, then centrifuged at 2190 g for 10 min (Eppendorf Centrifuge 5804/5804 R, Fisher Scientific, Segrate, Italy). Free hemoglobin (Hb) in the supernatant was measured using the HemoCue Plasma/Low Hb instrument (HemoCue AB, Kuvettgatan, Sweden). Hemolysis was calculated using the formula: Free HB x (100 – HCT CB-RBC) / Hb CB-RBC, with the result required to be < 0.8%, as specified by current regulations^28^.

### Irradiation

Irradiation was performed with a total dose of 30 Gy using an IBL 437 C irradiator for blood products and biological samples (CIS Bio International, Saclay, France), operating at a nominal dose rate of approximately 4–5 Gy/min. Samples were irradiated at room temperature on both D0 and D10 of storage. After irradiation, samples were maintained at 4 °C during transport between laboratories, and assays were initiated immediately upon arrival to minimize post-irradiation alterations. On D10, a new aliquot was drawn from the original storage bag and irradiated under identical conditions, simulating the irradiation of a blood unit immediately prior to transfusion.

### Ektacytometric analysis

The ektacytometric analysis was performed on Laser-assisted Optical Rotation Cell Analyzer (LoRRcaMaxSis, Mechatronics, Hoorn, The Netherlands), measuring the de-formability of RBC suspended in a polyvinylpyrrolidone (PVP) solution. During the test, a laser beam was directed at the RBC, causing them to emit a diffraction pattern, which was captured by a camera and converted into a curve using Osmoscan. The following parameters were evaluated: the Omin-value corresponding to the osmolality at which the deformability reaches its minimum and represents the 50% of the RBC hemolysis in conventional osmotic fragility assays; the elongation index (EI) max that corresponds to the maximal deformability and is an expression of the membrane surface; the Ohyper (the osmolality in the hypertonic region corresponding to 50% of the EImax) that reflects mean cellular hydration status^14,28^.

### Determination of ATP levels

ATP determination was carried out on perchloric acid extracts (PCA) using a spectrophotometric assay with an automated Kontron 930 instrument (Kontron Instruments Inc., Boston, MA, USA)^29^. A sample of RBC was prepared by adding 0.5 mL of 20% perchloric acid (Merck KGaA, Darmstadt, Germany) to 1 mL of blood. The mixture was kept on ice for 10 min, then centrifuged at 1200 g for 10 min at 4 °C (Sigma, Osterode am Harz, Germany). The supernatant was separated, and the volume noted. 50 ul of 0.05% Methyl orange solution was added to PCA and neutralized with 3 M potassium carbonate (Carlo Erba Reagents srl, Milano, Italy) under continuous stirring, until the color changed from pink to yellow. ATP concentration was measured at 340 nm, and calculated using the formula:

[ATP] = [(OD – OD_blank)/6.22] X (Fd × 100/Hb), Where: OD = Optical Density of the sample OD_blank = Optical Density of the blank Fd = Dilution factor (9.75). The values obtained were expressed in µmol/g Hb with reference values of [ATP] 3.6–4.9 µmol/g Hb.

### Morphological RBC analysis

Morphological analysis of RBC was performed using the May-Grünwald Giemsa (MGG) staining technique. Blood smears were prepared on clean glass slides and air-dried. Slides were fixed in methanol for 3 min and then stained sequentially with May-Grünwald solution for 5 min, followed by Giemsa stain (diluted 1:10 in buffered water, pH 6.8) for 15 min. After staining, slides were rinsed with distilled water, air-dried, and observed under optical microscopy (Axiophot system, Carl Zeiss Italia S.p.A) at 63x or 100x magnification using immersion oil. The morphology of RBC was evaluated for shape abnormalities (poikilocytosis), size variation (anisocytosis), membrane integrity, and presence of inclusions or immature forms.

### Statistical analysis

Results are analysed by descriptive statistics and presented as mean ± standard deviation SD or median, as specified in each figure legend. Statistical P values were calculated by Nonparametric Mann-Whitney test, as reported in figure legends, using GraphPad Prism 5 (Graphpad, San Diego, CA, USA). Where not differentially specified, *P < 0.05, **P < 0.01, ***P < 0.001, while *P* > 0.05 are not reported.

## Supplementary Information

Below is the link to the electronic supplementary material.

Supplementary Material 1

## Data Availability

All data generated or analysed during this study are included in this published article (and its Supplementary Information files).
