# Supplementary material for: Rheological and biochemical comparison of cord and adult blood red cells for transfusion applications
Source: Sci Rep. 2026 Mar 13;16:13320. doi: 10.1038/s41598-026-42457-4 (PMC13106815; doi:10.1038/s41598-026-42457-4)
Supplement: Supplementary file 1 — Supplementary Material 1 [file 41598_2026_42457_MOESM1_ESM.docx]

**Supplementary Materials:**


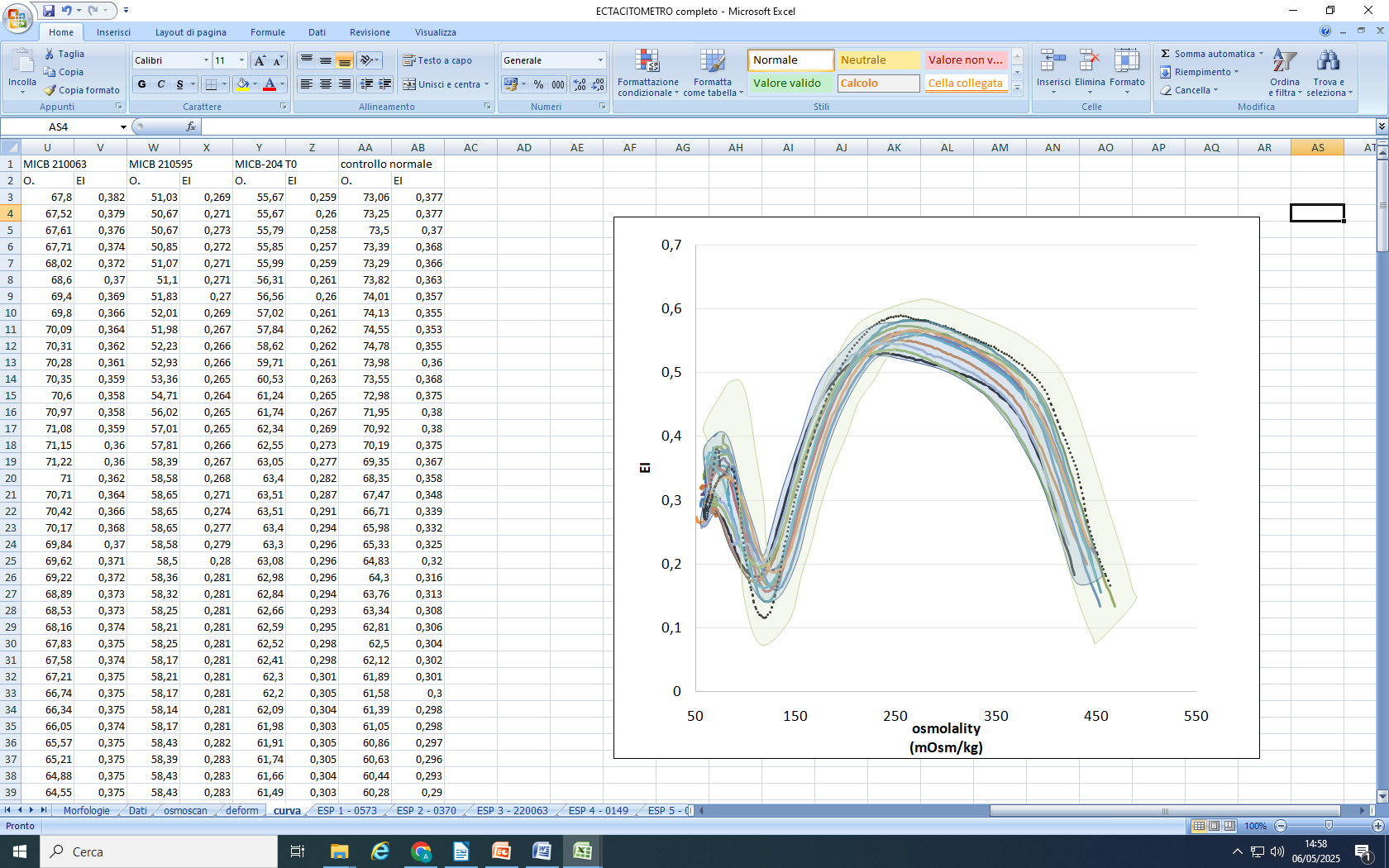


**Figure S1.** Established Reference Values for CB Samples in Ektacytometry

**Table S1.** Red Blood Cells morphology of whole adult and cord blood before and after irradiation and 10 days storage. SD: standard deviation

| **MORPHOLOGY ADULT WHOLE BLOOD STORED IN BAG** | | | | | | | | | | | | | | | | |  |  |
| --- | --- | --- | --- | --- | --- | --- | --- | --- | --- | --- | --- | --- | --- | --- | --- | --- | --- | --- |
| **TOTAL SAMPLES: 5** | **DAY 0** | | | | **DAY0 IRRADIATED** | | | | **DAY 10** | | | | **DAY10 IRRADIATED** | | | |  |  |
|  | **N** | **MEAN**  **%** | **DS** | **MEDIAN** | **N** | **MEAN**  **%** | **DS** | **MEDIAN** | **N** | **MEAN**  **%** | **DS** | **MEDIAN** | **N** | **MEAN**  **%** | **DS** | **MEDIAN** |  |  |
| **ECHINOCYTES** | 5 | 7 | 3.9 | 7 | 5 | 11 | 3.8 | 12 | 4 | 25.7 | 12.2 | 27 | 4 | 24 | 14.5 | 23 |  |  |
| **SPHEROCYTES** | 3 | 4.3 | 2.3 | 3 | / | / | / | / | 3 | 5.7 | 2.5 | 6 | 3 | 5.3 | 1.5 | 5 |  |  |
| **OVALOCYTES** | 5 | 4.2 | 1.3 | 4 | 5 | 5.2 | 2.3 | 5 | 3 | 4.3 | 1.2 | 5 | 3 | 5 | 1.7 | 4 |  |  |
| **SCHISTOCYTES** | / | / | / | / | 3 | 3.7 | 0.6 | 4 | / | / | / | / | / | / | / | / |  |  |
| **STOMATOCYTES** | 1 | 5 | 0 | 5 | 1 | 3 | 0 | 3 | / | / | / | / | / | / | / | / |  |  |
| **DACROCYTES** | / | / | / | / | / | / | / | / | / | / | / | / | / | / | / | / |  |  |
| **MORPHOLOGY WHOLE CORD BLOOD STORED IN BAG** | | | | | | | | | | | | | | | | |  |  |
| **TOT SAMPLES: 12** | **DAY 0** | | | | **DAY0 IRR** | | | | **DAY 10** | | | | **DAY10 IRR** | | | |  |  |
|  | **N** | **MEAN**  **%** | **DS** | **MEDIAN** | **N** | **MEAN**  **%** | **DS** | **MEDIAN** | **N** | **MEAN**  **%** | **DS** | **MEDIAN** | **N** | **MEAN**  **%** | **DS** | **MEDIAN** |  |  |
| **ECHINOCYTES** | 10 | 5.9 | 3.1 | 4.5 | 11 | 7 | 3.4 | 7 | 12 | 23 | 8.5 | 24.5 | 12 | 19.9 | 7.2 | 17.5 |  |  |
| **SPHEROCYTES** | 12 | 4 | 2.5 | 3 | 11 | 3.4 | 1.3 | 3 | 12 | 6.3 | 5.9 | 4.5 | 12 | 5.8 | 1.9 | 5.5 |  |  |
| **OVALOCYTES** | 2 | 2 | 0 | 2 | 1 | 4 | 0 | 4 | 3 | 3 | 1 | 3 | 5 | 2.6 | 0.5 | 3 |  |  |
| **SCHISTOCYTES** | 5 | 2.2 | 0.8 | 2 | 4 | 3.5 | 2.1 | 3.5 | 2 | 4.5 | 2.1 | 4.5 | 2 | 4.5 | 0.7 | 4.5 |  |  |
| **STOMATOCYTES** | 5 | 4.8 | 2.0 | 4 | 5 | 4 | 1.9 | 5 | 4 | 3.5 | 1.3 | 3.5 | 3 | 4 | 0 | 4 |  |  |
| **DACROCYTES** | 1 | 2 | 0 | 2 | 1 | 2 | 0 | 2 | 2 | 3 | 1,4 | 3 | 1 | 6 | 0 | 6 |  |  |
